# Sheets of Vertically Aligned BaTiO_3_ Nanotubes Reduce Cell Proliferation but Not Viability of NIH-3T3 Cells

**DOI:** 10.1371/journal.pone.0115183

**Published:** 2014-12-15

**Authors:** Marianna Giannini, Martina Giannaccini, Teresa Sibillano, Cinzia Giannini, Dun Liu, Zhigang Wang, Andrea Baù, Luciana Dente, Alfred Cuschieri, Vittoria Raffa

**Affiliations:** 1 Institute of Life Science, Scuola Superiore Sant'Anna, Pisa, Italy; 2 Institute of Crystallography, National Research Council, (IC-CNR), Bari, Italy; 3 Institute for Medical Science and Technology, University of Dundee, Dundee, United Kingdom; 4 Department of Biology, Università di Pisa, Pisa, Italy; Osaka University, Japan

## Abstract

All biomaterials initiate a tissue response when implanted in living tissues. Ultimately this reaction causes fibrous encapsulation and hence isolation of the material, leading to failure of the intended therapeutic effect of the implant. There has been extensive bioengineering research aimed at overcoming or delaying the onset of encapsulation. Nanotechnology has the potential to address this problem by virtue of the ability of some nanomaterials to modulate interactions with cells, thereby inducing specific biological responses to implanted foreign materials. To this effect in the present study, we have characterised the growth of fibroblasts on nano-structured sheets constituted by BaTiO_3_, a material extensively used in biomedical applications. We found that sheets of vertically aligned BaTiO_3_ nanotubes inhibit cell cycle progression - without impairing cell viability - of NIH-3T3 fibroblast cells. We postulate that the 3D organization of the material surface acts by increasing the availability of adhesion sites, promoting cell attachment and inhibition of cell proliferation. This finding could be of relevance for biomedical applications designed to prevent or minimize fibrous encasement by uncontrolled proliferation of fibroblastic cells with loss of material-tissue interface underpinning long-term function of implants.

## Introduction

Barium titanate (BaTiO_3_) belongs to the group of ferroelectric ceramics. It is characterized by high dielectric constant and high Curie temperature [1]. Because of its interesting physical properties and superior biocompatibility confirmed by both *in vitro* [2]–[4] and *in vivo* studies [5], [6], BaTiO_3_ has been investigated for various applications in tissue engineering. Its unique mechanical properties, including the ability to form strong mechanical interfacial bonds with tissues [5] and the strong piezoelectric behaviour following electrical poling [7], has enabled the successful use and testing of BaTiO_3_ both *in vitro* and *in vivo* as medical implants for osseo-integration. *In vitro* studies have demonstrated that negatively and positively poled BaTiO_3_ enhance the formation of bone-like crystals, such as calcium phosphate. Although the underlying mechanism remains unknown, it has been suggested that, depending on the poling direction, a negatively or positively charged surface could attract positive or negative ions, respectively, which behave as nuclei for the formation of bone-like crystal growth [8]–[10]. The capability of the poled BaTiO_3_ to enhance the formation of such crystals could explain the results of several *in vivo* implantation studies with BaTiO_3_ based grafts [11], [12], in which improved osteogenesis and bone formation around the implant were observed. Furthermore, charged surfaces could drive preferential absorption of proteins, through electrostatic attraction of protein charged groups [13]. This could explain the bioactivity of poled BaTiO_3_ and, in particular, its ability to improve cell proliferation *in vitro* [4].

Other studies have indicated that unpoled BaTiO_3_ can also function as a bioactive material. Thus, it has been demonstrated *in vitro* that unpoled BaTiO_3_ enhances cell metabolism to the same extent as the poled material [14]. This observation suggests that mechanisms, different from the superficial charge, such as material topography, chemistry and structure, could also account for these biological effects.

With recent technological advances in materials science, molecular cell biology and nanotechnology, attention is increasingly being focused on the study of the functional advantages of nano-structured materials, at the cellular and molecular levels, for biomedical applications. The biological responses of nano-structured surfaces are different from that of the bulk material, because nano-structuration confers a much larger surface area per unit of mass, thereby increasing chemical reactivity [15].

The aim of this study is to explore the biological effects of sheets of BaTiO_3_ nanotubes as a novel implantable material able to drive specific cellular responses and, more specifically, to gain control on processes that naturally occur when foreign materials are implanted in the human body. In particular this study targets fibroblasts, which are stimulated to proliferate and to deposit the connective tissue during a process of fibrosis [16], and explores potential mechanisms that could impair this phenomenon. Recently, anodic aluminium oxide (AAO) membranes have been used for template-assisted growth of arrays of vertically aligned nanotubes (VANTs). Different methodologies have been developed to synthesize these unidimensional nano-structures. The most common approach is the sol–gel electrophoretic deposition (EPD), which is based on filling the AAO template membrane with starting sol particles using an electric potential [17]. In the present study, we used a protocol developed by Chen et al., which produces VANTs of BaTiO_3_ in AAO membranes by using a mild process at near-ambient conditions without the application of heat treatment, external electric fields, or pre-existing ceramic particles [18]. Despite the numerous reports on the synthesis of BaTiO_3_ nanotubes, the characterization of their biological behaviour remains unknown. Here we demonstrate that, even if the nanotube material is not poled and not crystalline, AAO membranes filled with VANTs of BaTiO_3_ clearly induce a specific biological response. Specifically, we observed in the embryonic fibroblast NIH-3T3 cell line that the nano-structured material influences the cell cycle by decreasing the rate of cell proliferation, without affecting cell viability. Because of the extensive use of BaTiO_3_ in tissue engineering, our findings could represent a strategy to be explored for improvement in the overall performance of such implants by abrogation of the fibrous encapsulation. In particular, our work suggests that surface nano-structuration of BaTiO_3_ could be investigated as a strategy to reduce the fibrosis which naturally occurs around implanted materials due to the uncontrolled proliferation of fibroblast cells around the implantation site.

## Materials and Methods

### Synthesis of BaTiO_3_ nanotubes

Arrays of BaTiO_3_ VANTs were synthesized on anodic aluminium oxide (AAO) templates [18]. AAO templates are commercially available filtration membranes (Whatman, Anodisc, diameter 47 mm or 13 mm, thickness 60 µm; pore diameter 200 nm).

Briefly, ammonium hexafluorotitanate ((NH_4_)_2_TiF_6_ 10 mM, Sigma-Aldrich Co) and barium nitrate (Ba(NO_3_)_2_ 10 mM, Sigma-Aldrich Co) were dissolved in aqueous solution of boric acid (30 mM, Sigma-Aldrich Co) at room temperature. The pH was adjusted to 2.0 by adding 6 M HCl drop wise. The AAO membranes were vertically immersed in the precursor solution and held at 60°C in a bath for 20 h. The membranes were then removed from the solution and rinsed with deionized water for 5 min (2 times) and with phosphate buffered solution (PBS) for 5 min (2 times). As control group, a non nano-structured (NNS) layer of BaTiO_3_ was deposited by using as template material a round glass coverslip (diameter 13 mm), processed as described for AAO membranes.

The AAO membrane naked and filled with the BaTiO_3_ nanotubes and the glass coverslip coated with BaTiO_3_ will be hereafter labelled as AAO, AAO-NT and NNS, respectively.

### Scanning electron microscopy (SEM) and micro-analysis

Electron imaging was performed with a scanning electron microscope (FEI XL20) equipped with Energy Dispersive X-ray spectrometer (EDX, EDAC model). SEM analysis was used in order to obtain information about the morphology. EDX microanalysis allowed a chemical mapping at microscopic level. For cell imaging, after 48 h of incubation, the cells were washed with PBS, fixed with formaldehyde 4% for 15 min, dehydrated via 5 min immersions in increasing concentrations of methanol 30% (x2), 50% (x2), 70% (x2) and 90% (x2), followed by further dehydration with anhydrous methanol and allowed to dry overnight at room temperature. For SEM imaging, the samples were sputtered with 20 nm of gold.

### Magnetic Force Microscopy (MFM)

AAO, AAO-NT, NNS and cell culture plates (hereafter labelled as P) were placed onto the atomic force microscopy (AFM) stage and imaged using ScanAsyst Adaptive mode on the Bioscope Catalyst (Bruker). Samples were also analysed on MFM mode [19] and magnetic coated tips were used (PPP-MFMR-10, Nanosensors – resonance frequency 45–115 kHz, spring constant 0.5–9.5 Nm^−1^) (tip distance from surface: 80 nm). Roughness was measured via the Nanoscope Analysis Software on 4×4 µm^2^ areas (unless stated otherwise in the text).

### X-ray Powder Diffraction

X-ray diffraction data were collected at room temperature from AAO-NT and AAO. Commercial crystalline BaTiO_3_ nanoparticles (spherical 200 nm particles with tetragonal structure and 99.9% purity) deposited onto silicon substrates were used as control groups (1148DY, Nanostructured & Amorphous Materials, Inc.). Measurements were performed by a Bruker D8 Discover diffractometer, equipped with a Göbel mirror, using Cu Kα radiation (λ_Kα1_ = 1.54056 Å and λ_Kα2_ = 1.54439 Å), and a scintillation detector. The working conditions were set to 40 kV and 50 mA. Data were collected at fixed incidence angle of 5° while moving the detector in the range 10–80° with a step size of 0.05°. As a consequence, a penetration depth of several tens of micrometers in aluminium oxide was probed.

### Cell cultures

The NIH-3T3 murine fibroblast cell line (ATCC) was cultured at 37°C with 5% CO_2_ in Dulbecco's Modified Eagle's Medium (DMEM) containing 10% heat inactivated foetal bovine serum (FBS), 2 mM L-glutamine, 100 IU/ml penicillin, 100 µg/ml streptomycin and 0.75 µg/ml amphotericin-B. Poly-L-lysine (PLL) coating was performed by incubating cell culture plates (P) at 37°C for 1 h with PLL (Sigma-Aldrich Co) 10 µg/ml in PBS. Similarly, before cell seeding, AAO or AAO-NT or NNS substrates were placed on the bottom of the wells of 6 or 12 well plates and coated with PLL as described. 24 h after cell seeding, AAO or AAO-NT or NNS substrates were placed on the bottom of new wells, in order to exclude from the analysis cells adhering on the bottom of the well. The coated substrates are hereafter labelled with PLL superscript (e.g., P^PLL^, AAO^PLL^, AAO-NT^PLL^ and NNS^PLL^).

### Cell viability and apoptosis

Propidium iodide (PI, Sigma-Aldrich Co) dye exclusion assay was used to assess cell viability. Hoechst 33342 (Sigma-Aldrich Co) staining was used to distinguish condensed apoptotic (pyknotic) nuclei.

5·10^5^ cells were seeded in 6 cm Petri dishes on P, P^PLL^, AAO^PLL^, AAO-NT^PLL^ and NNS^PLL^ and incubated for 72 h. Then Hoechst 33342 was added at a final concentration of 5 µg/ml and incubated for 10 min at 37°C. Then PI was added at the final concentration of 2.5 µg/ml and incubated for 5 min at 37°C. For each experiment, 6 replicates have been performed and an average of 2000 cells per experiment was counted. Microscopy and digital image acquisitions were carried out by using a Nikon eclipse TE2000-U fluorescent microscope equipped with Nikon Digital Sight DS-U2 camera. For image acquisition NIS Elements imaging software was used.

### Phalloidin Staining

Cytoskeletal organization in adherent, cultured fibroblasts was observed as an indication of the relative degrees of cell spreading on various substrates. For this purpose F-actin was stained by fluorescently-tagged phalloidin (R415, Life Technologies). Fibroblasts (2.5·10^4^ cells) were seeded in 24-well plate on P, P^PLL^, AAO, AAO^PLL^, AAO-NT, AAO-NT^PLL^ and glass cover slips (negative control). Cells were cultured for 72 h before carrying out F-actin staining. The culture medium was removed, cells were gently washed with PBS and, then, fixed with formaldehyde 4% for 15 min. After washing, cells were permeabilized with 0.1% Triton X-100 in PBS and blocked with 10% foetal bovine serum and 0.1% Triton X-100 in PBS for 1 h at room temperature. Cells were sequentially incubated with Phalloidin-Rhodamine for 3 h at room temperature, stained for Hoechst 33342 (5 µg/ml) for 10 min and rinsed in PBS. The images were analysed by fluorescent microscopy and the areas were measured using ImageJ software. The experiment was conducted in triplicate and an average of 300 cells per experiment was measured.

### Cell proliferation

Fibroblasts (2.5·10^4^ cells) were seeded in 24-well plate on P, P^PLL^, AAO^PLL^, AAO-NT^PLL^ and NNS^PLL^. Proliferation was analysed in epifluorescence by using rabbit polyclonal anti-phospho-histone H3 immunostaining (Upstate Biotechnology). After 72 h of incubation the immunocytochemistry protocol was performed. The culture medium was removed, the cells were gently washed with PBS and, then, fixed with formaldehyde 4% for 15 min. After washing, cells were permeabilized with 0.1% Triton X-100 in PBS and blocked with 1% bovine serum albumin in PBS for 1 h. Then the cells were incubated with polyclonal anti-phospho-histone H3 for 3 h at room temperature, washed in PBS, incubated with Rhodamine goat-anti rabbit secondary antibody (Life Technologies) for 1 h at room temperature, stained for Hoechst 33342 (5 µg/ml) for 10 min and rinsed in PBS. Cell counting was conducted by fluorescence microscopy analysis. Six experimental replicates were performed and for each experiment an average of 1600 cells were counted.

Cell proliferation was also studied using Click-iT EdU imaging kit (Life Technologies), which marks DNA synthesizing cells. After 72 h incubation, cells reached 60% confluence and Click-iT EdU protocol was performed, following the producer instructions. Cells were fixed and EdU revelation with Alexa fluor azide (provided in the kit) was made 8 h after the addition of the EdU to the cell culture medium. Cells were then stained for Hoechst 33342 (5 µg/ml) for 10 min and rinsed in PBS.

Cell counting was performed by fluorescence microscopy analysis. The experiment was conducted in 6 replicate and for each experiment a minimum of 3000 cells was counted.

### Cell detachment assay

In order to evaluate the strength of cell attachment to the substrates (P, P^PLL^, AAO^PLL^, AAO-NT^PLL^ and NNS^PLL^), cells were stained with Hoechst 33342 (5 µg/ml) for 10 min, treated with EDTA 50 mM and the Petri dishes were placed to float in a sonication bath for 10 minutes (230 V, 80 W, 37 kHz, Elma). After that, cells were washed twice with PBS. Hoechst stained nuclei images were recorded at fixed position before and after the EDTA/sonication experiment. Six experimental replicates were conducted and for each experiment cell decrement was evaluated, starting from an initial number of 3000 cells.

*Statistical analysis*

Values are reported as the mean ± standard error of the mean. We studied the distributions of the data by Kolmogorov-Smirnov test. Statistical significance was assessed by one-way analysis of variance. Specifically, for non-normal data distributions, Kruskal-Wallis analysis was used, followed by multi-compare analysis (95% confidence), whereas for normal data distributions, we used ANOVA followed by Bonferroni correction. Significance was set at p≤0.05. “*” is the significance vs the P group, “#” is the significance vs the P^PLL^ group, “§”is the significance vs the NNS^PLL^ group, “n.s.” indicates non-significance. Statistical analyses were performed in Matlab R14 workspace (functions “kstest”, “anova1”, “bonferroni”, “multicompare”) and Microsoft Office Excel using data analysis tool.

## Results

### Synthesis and characterization of BaTiO_3_ nanotubes

Morphology and chemical composition of BaTiO_3_ nanotubes synthesized on AAO templates were characterized using different approaches.

**Figure 1:**
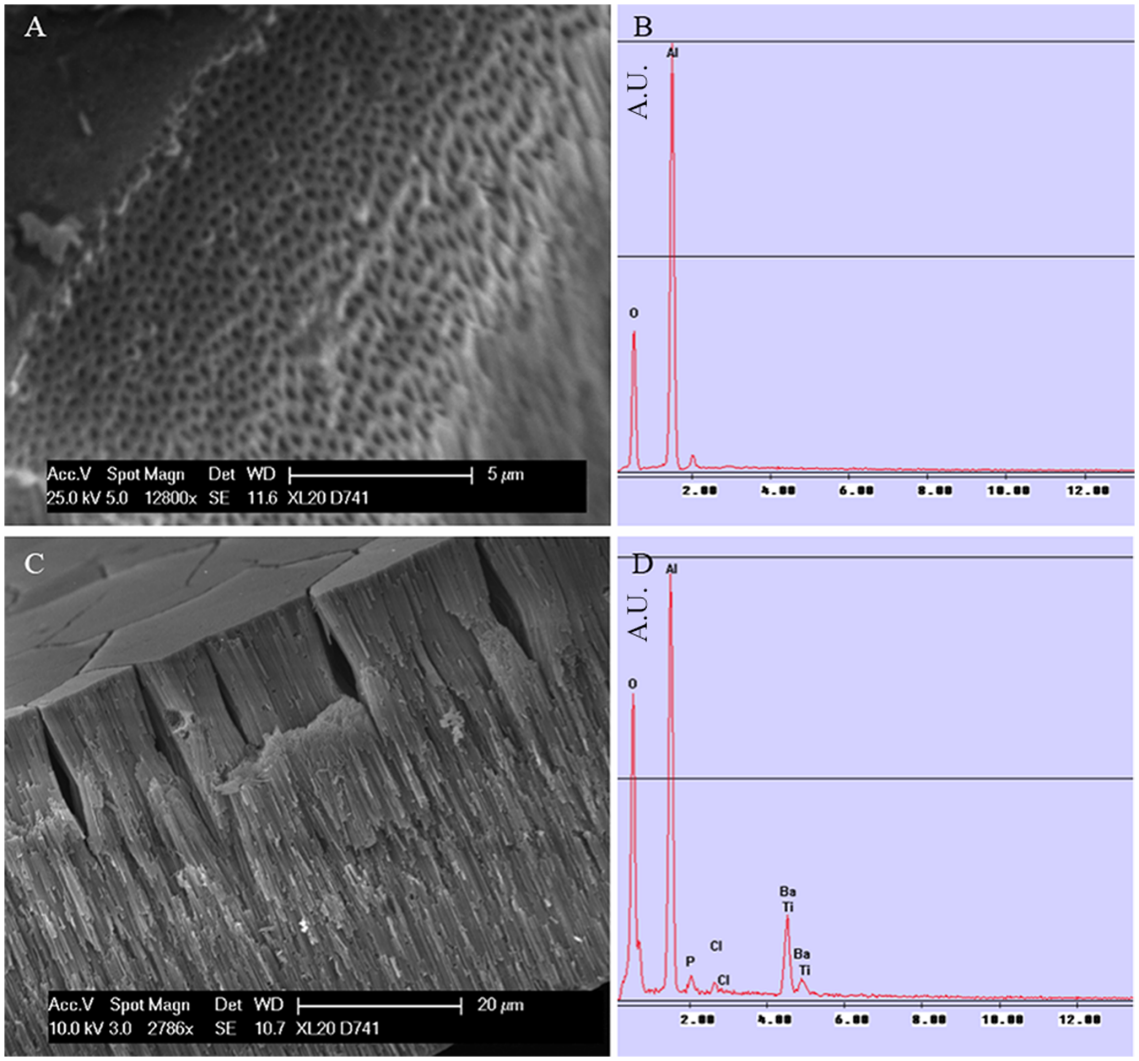
SEM (A) and EDX (B) of AAO. SEM (C) and EDX (D) of AAO-NT.

SEM microscopy of transverse sections of AAO-NT shows the presence of vertically aligned tubular nano-structures (fig. 1, C). The EDX spectrum confirmed that AAO-NT contains Ba and Ti (from nanotubes) and Al from template membrane (fig. 1, D). In contrast, in AAO control membrane (fig. 1, A) Al was detected, but Ba and Ti were not (fig. 1, B). The small amount of P and Cl detected were derived from the PBS wash after the synthesis.

**Figure 2:**
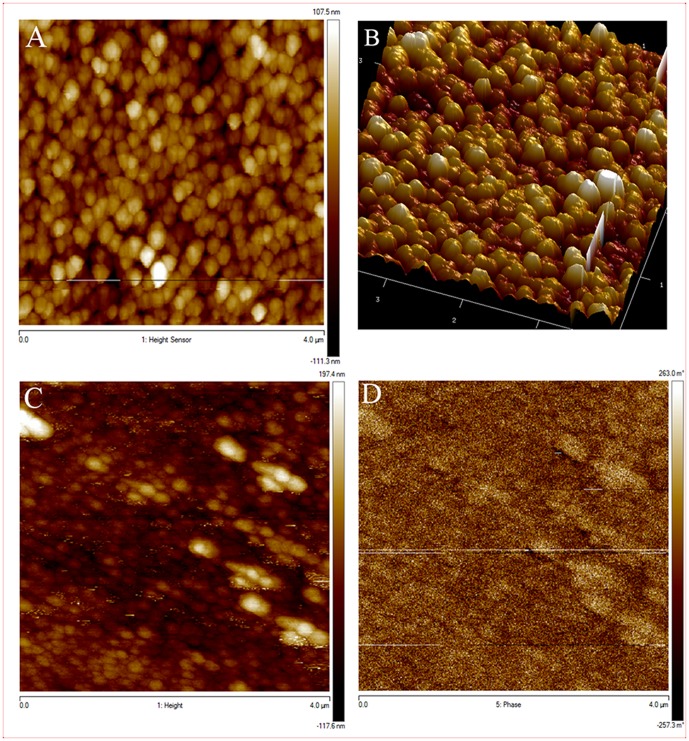
AFM reconstruction of 2D (A) and 3D (B) morphology of AAO-NT. MFM height image (C) and phase image (D). Scale in µm.

AFM 2D and 3D reconstructions of the AAO-NT surface morphology (fig. 2, A–B) confirm that the circular holes of the template are filled with nanotubes. In the white pixels area on MFM height image (fig. 2, C) a phase difference was experienced (fig. 2, D), due to an external magnetic force applied onto the cantilever, as the tip is magnetically coated. This suggests that the nanotubes have a magnetic moment.

**Figure 3:**
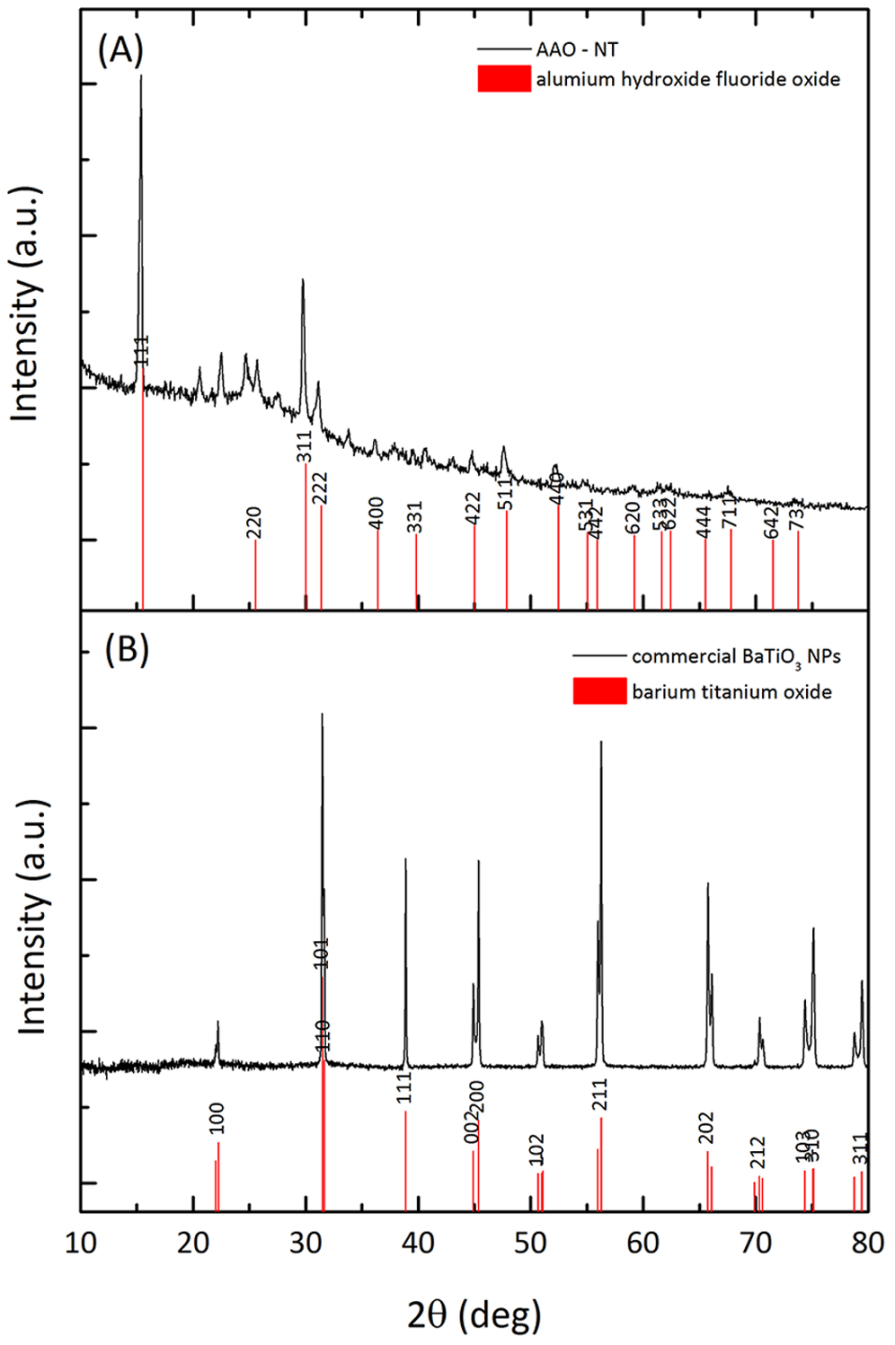
XRD spectra recorded from the AAO-NT (A) and crystalline (tetragonal structure) BaTiO_3_ NP (B).

VANTs synthesized by template-assisted process were initially examined by X-ray powder diffraction (XRD). XRD spectra were recorded from the AAO-NT (fig. 3, A) and crystalline (tetragonal structure) commercial BaTiO_3_ NP (fig. 3, B). The XRD spectrum in fig. 3, A revealed peaks that were indexed as the aluminium hydroxide fluoride hydrate crystalline phase. Unindexed peaks were ascribed to residual impurities resulting from the synthesis process. No crystalline BaTiO_3_ in the AAO-NT sample was detected, while an amorphous phase can be revealed in the spectrum which could be assigned to a mixed contribution coming from AAO amorphous substrate and amorphous BaTiO_3_ phase [20]. The absence of the annealing step of the sample inside AAO after the synthesis probably inhibits the change into crystalline BaTiO_3_ [20].

### Cell spreading

**Figure 4:**
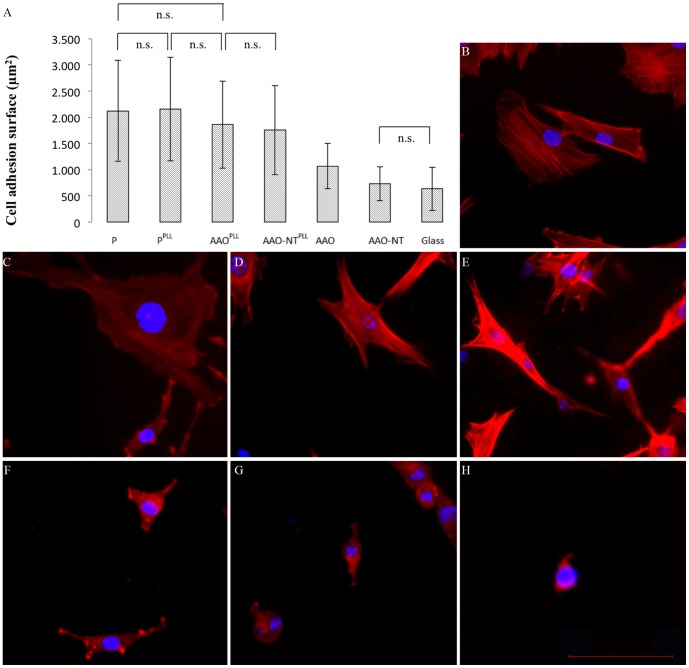
Cell surface area. N = 3. Kruskal-Wallis followed by multicompare analysis, p = 0. Actin (red) and nuclei (blue) staining on P, P^PLL^, AAO^PLL^, AAO-NT^PLL^, AAO, AAO-NT and glass, respectively (B–H). Scale bar: 100 µm.

As the scaffold and the coating could affect cell adhesion and surface spreading, we measured the surface area of cells seeded on non-coated (P, AAO, AAO-NT) and PLL coated (P^PLL^, AAO^PLL^, AAO-NT^PLL^) substrates (fig. 4). Glass coverslips were used as control of poor cell adhesion. Results showed that the presence of the PLL does not change surface adhesion on cell culture plates (P, P^PLL^) but drastically changes cell spreading on AAO/AAO^PLL^ and AAO-NT/AAO-NT^PLL^. In particular cell spreading on AAO-NT was found similar to that on glass coverslip, confirming the poor cell adhesion on non-coated AAO-NT. Interestingly, cell adhesion on AAO^PLL^ (cell adhesion surface 1859±832 µm^2^) was not found different from the controls (P^PLL^, cell adhesion surface 2157±991 µm^2^, and P, cell adhesion surface 2124±968 µm^2^) and the membrane filled with the VANTs (AAO-NT^PLL^, cell adhesion surface 1755±848 µm^2^). Based on these data, substrate coating is required for good cell spreading.

### Cell morphology

**Figure 5:**
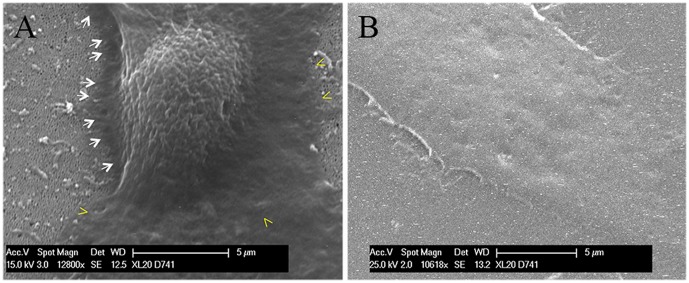
SEM imaging of NIH-3T3 cells on AAO-NT^PLL^ (A) and P^PLL^ (B). Arrows and arrowheads show cell interactions with the substrate.

Cell morphology was examined by SEM which confirmed that cells seeded on AAO-NT^PLL^ exhibit a normal morphological phenotype (fig. 5, A), indistinguishable from the phenotype of cells seeded on AAO^PLL^ (not shown) or P^PLL^ (fig. 5, B). Despite cell spreading looks similar in all samples (in agreement to data on cell surface area provided in fig. 4), electron microscopy showed that cells adherent on AAO-NT^PLL^ are not just flattened on the surface as in the controls (fig. 5, B) but are firmly interconnected with the substrate (fig. 5, A, yellow arrowheads) and cellular processes seems to be under tension due to the strong interactions with the VANTs (fig. 5, A, white arrows).

### Cell proliferation

**Figure 6:**
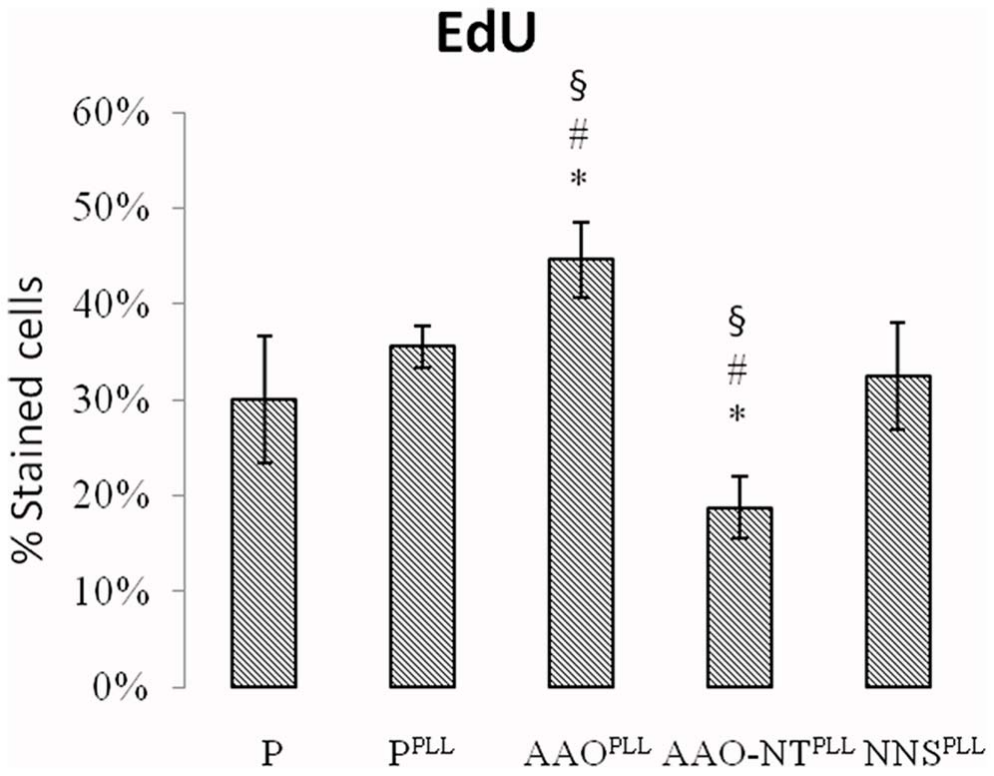
Percentage of cells positive to EdU on P, P^PLL^, AAO^PLL^, AAO-NT^PLL^ and NNS^PLL^. N = 6. ANOVA followed by Bonferroni analysis, p = 2·10^−8^.

Cell proliferation was studied using the EdU (5-Ethynyl-2′-deoxy-uridine) click assay, by counting the number of positive cells on P, P^PLL^, AAO^PLL^, AAO-NT^PLL^ and NNS^PLL^ after 8 h of incubation with the EdU reagent. Marked cells were found to be 30.07±6.63% in P, 35.55±2.13% in P^PLL^, 44.63±3.88% in AAO^PLL^, 18.72±3.23% in AAO-NT^PLL^ and 32.45±5.63% in NNS^PLL^. Data analysis showed that the percentage of DNA synthesizing cells was statistically different between AAO-NT^PLL^ and both control groups (P, P^PLL^). This decrease of cell proliferation could not be ascribed to the presence of the membrane (AAO^PLL^ is characterized by an increase of cell proliferation which is statistically significant compared to both controls P and P^PLL^) as well as the material itself (NNS^PLL^ is not statistically different from both controls P and P^PLL^) (fig. 6).

Proliferation was also measured counting the number of cells positive to the phospho-histone H3 immunostaining, as histone H3 is specifically phosphorylated during both mitosis and meiosis, when metaphase chromosomes are heavily phosphorylated [21]. Mitotic cells were found to be 13.78±3,81% in the P, 11.67±2,26% in AAO^PLL^ and 5.94±1,36% in AAO-NT^PLL^, confirming a decrease of cell proliferation in AAO-NT^PLL^ sample which was statistically different from both AAO^PLL^ and the control group P.

### Cell viability and apoptosis

**Figure 7:**
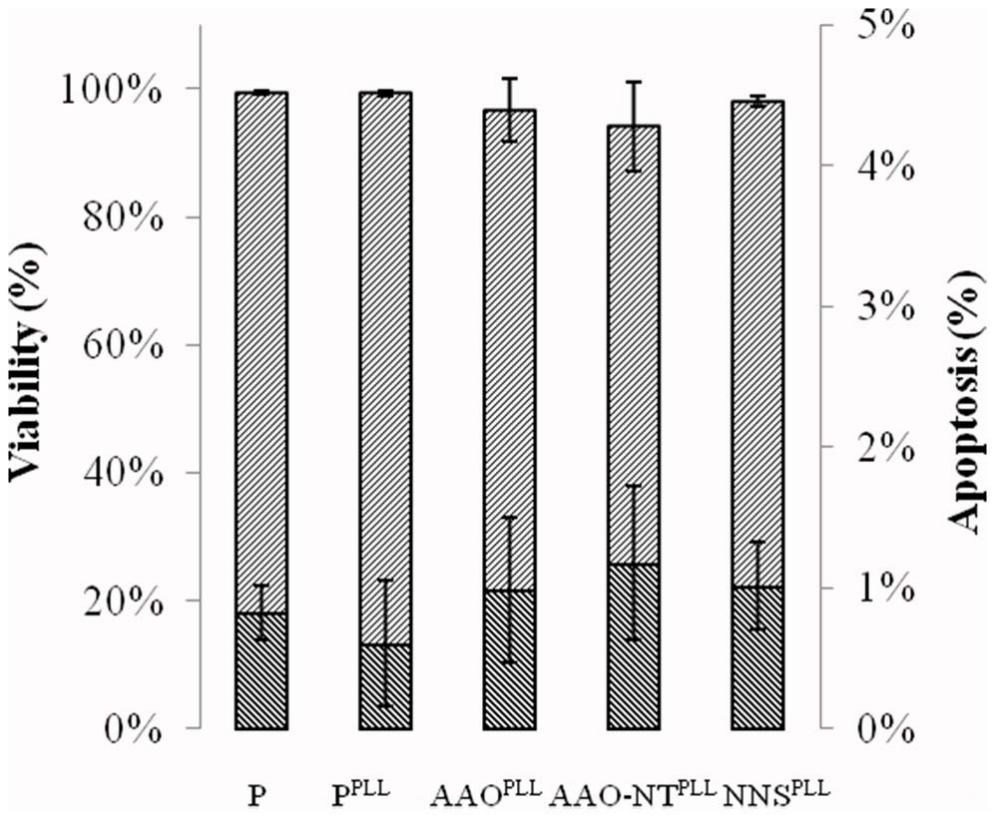
Viability and apoptosis of NIH-3T3 on P, P^PLL^, AAO^PLL^ and AAO-NT^PLL^ and NNS^PLL^. Cell viability (p = 0.087). Cell apoptosis (p = 0.309). N = 6. Kruskal-Wallis followed by multicompare analysis.

Cell viability was tested by using propidium iodide (PI) dye exclusion assay. Fluorescence microscopy analysis showed that the treatment induced a negligible toxicity after 72 h. Thus, the viability was 96.70±4.99% for cells seeded on AAO^PLL^, 94.10±6.99% for cells seeded on AAO-NT^PLL^ and 98.07±0.84% for cells seeded on NNS^PLL^, not dissimilar from controls P (99.43±0.19%) and P^PLL^ (99.28±0.50%). The viability among the groups was similar (p = 0.087) (fig. 7). Furthermore there was no statistical difference in apoptosis among the groups (p = 0.309), with 0.83±0.19% for P, 0.61±0.44% for P^PLL^, 0.99±0.51% for AAO^PLL^, 1.17±0.55% for AAO-NT^PLL^ and 1.02±0.31% for NNS^PLL^ (fig. 7).

### Cell detachment assay

**Figure 8:**
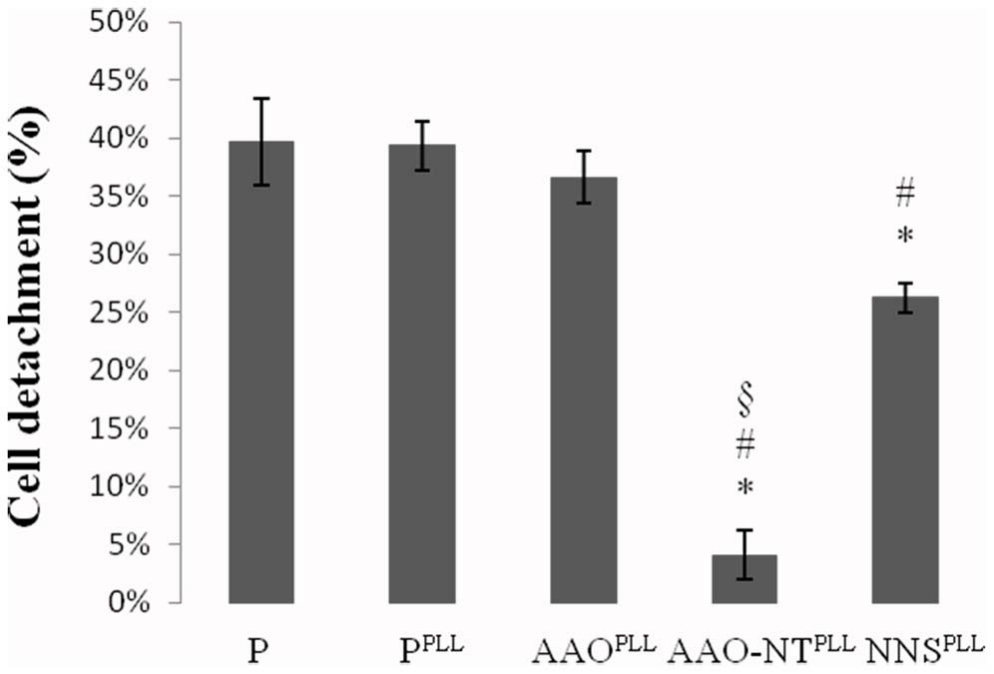
Cell detachment percentage after 50 mM EDTA and ultrasounds treatment on P, P^PLL^, AAO^PLL^, AAO-NT^PLL^ and NNS^PLL^, N = 6. ANOVA followed by Bonferroni analysis, p = 0.

Cells cultured on P, P^PLL^, AAO^PLL^, AAO-NT^PLL^ and NNS^PLL^ were mechanically detached from the substrates using ultrasounds and EDTA. When cells were cultured on AAO-NT^PLL^, mechanical detachment was very much lower (4.08±2.16%) compared to those on control groups P (39.69±3.69%), P^PLL^ (36.64±2.29%), AAO^PLL^ (36.64±2.29%) and NNS^PLL^ (26.25±1.24%) (fig. 8). AAO-NT^PLL^ and NNS^PLL^ are significantly different from all groups. No statistical difference among P, P^PLL^ and AAO^PLL^ was found.

## Discussion

We synthesized sheets of BaTiO_3_ VANTs, using an established protocol reported in the literature based on the growth of nanotubes in a nanoporous scaffold membrane [18]. The presence of VANTs in the membranes was confirmed by SEM (fig. 1, C), which showed the presence of nanotubular structures filling the nanopores of the template membranes. The EDX analysis confirmed that Ba and Ti - the elementary components of BaTiO_3_ - constitute such structures (fig. 1, D). AFM/MFM confirmed the presence of tubular structures coming out from the nanopores of the membranes (fig. 2, A–B) and also revealed that they exhibit weak magnetic properties (fig. 2, C–D). Additionally, the XRD powder diffraction, performed directly on the AAO-NT, demonstrated that VANTs were not constituted by crystalline BaTiO_3_ (fig. 3, A–B).

**Figure 9:**
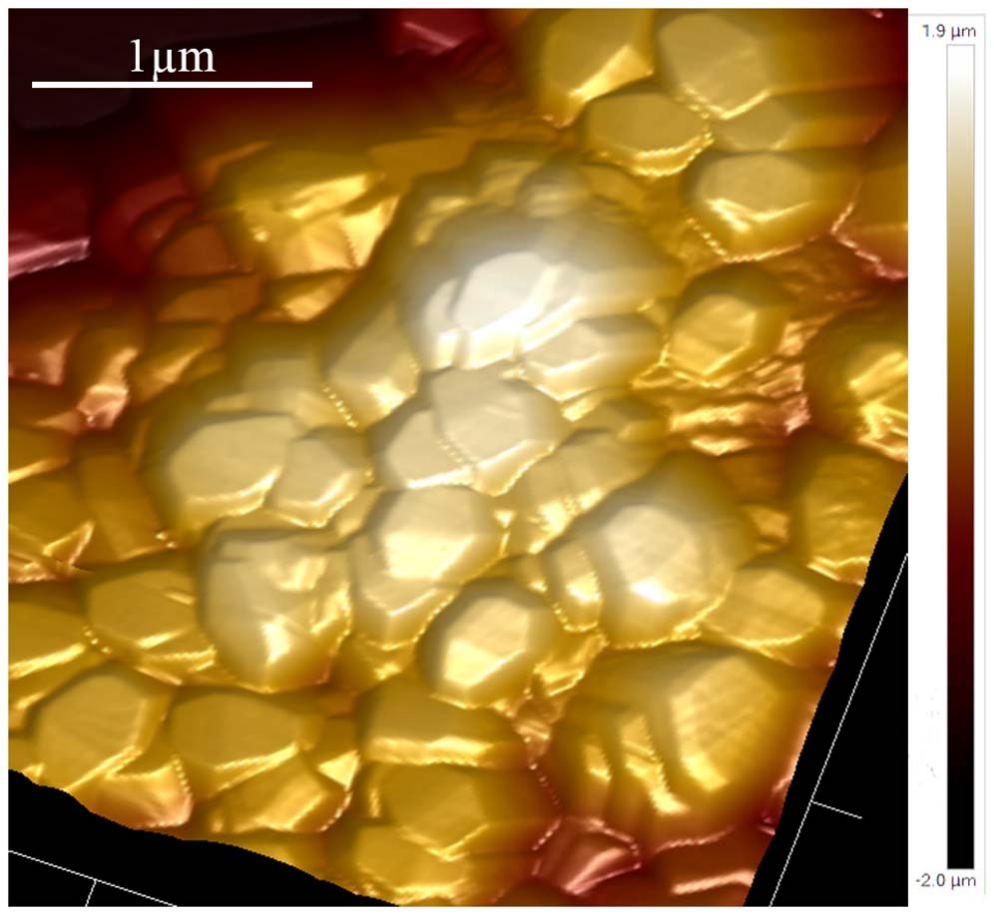
AFM 3D reconstruction of the morphology of NNS BaTiO_3_.

The main biological finding of the present study is that NIH-3T3 fibroblasts grown on BaTiO_3_ VANTs exhibit a decreased cell proliferation (fig. 6). Results obtained by 2 independent detection methods, the phospho-histone H3 staining and the EdU cell proliferation assay, show that the percentage of positive cells (i.e., the number of cells in active stage of replication) seeded on AAO-NT^PLL^ is approximately half of the positive cells cultured on controls (P and P^PLL^). Importantly, both assays confirmed that the reduced proliferation of fibroblasts is not related to the presence of the template material, as no significant difference in cell proliferation was detected between AAO^PLL^ and control P by phospho-histone H3 staining, while a slight increase of cell proliferation in AAO^PLL^ compared to controls P and P^PLL^ was observed by EdU staining. In order to exclude that the observed decrease of cell proliferation observed in AAO-NT^PLL^ is related to the material itself, a non nano-structured layer of BaTiO_3_ was deposited on a glass coverslip. AFM imaging of NNS confirms that this sample lacks of any nano-structuration, being organized in grains of variable size between half and few microns (fig. 9). Cell proliferation was assessed on cells seeded on NNS BaTiO_3_ but no statistically significant difference with controls (P and P^PLL^) was found. These data confirm that the observed decrease of cell proliferation result exclusively from the presence of the BaTiO_3_ nanotubes.

In speculating on the underlying mechanisms of this biological effect, the first consideration is to exclude that the decrease of cell proliferation is a cytotoxic effect of the nano-structured material itself. This can be dismissed as the VANTs were demonstrated to be non-cytotoxic to the NIH-3T3 fibroblasts, with no change in the viability of the cells seeded on AAO-NT^PLL^ and few apoptotic cells, below 1.5% in all groups (fig. 7). Cells seeded on VANTs displayed a normal nuclear morphology, as evidenced by Hoechst nuclear staining (data not shown). Once cytotoxicity effects were excluded, we investigated the possibility that nano-topography of the material could influence the cell cycle. In fact, several studies have demonstrated that surface nano-topography and roughness can influence cell morphology and adhesion [22]–[24].

To address the behaviour of cells seeded on the VANTs, we quantified cell spreading by mean of actin staining on the different substrates. The epifluorescence images obtained showed a comparable actin network in all groups (with the exception of cells grown on non coated substrates AAO, AAO-NT and glass) (fig. 4). Based on calculations of the cell surface area, we found that cell spreading on AAO-NT^PLL^ was not significantly different from cell spreading on the template material (AAO^PLL^), confirming that the decreased cell proliferation was not related to a decrease of cell spreading. To analyse in detail the nature and extent of the interactions between cells and the underlying substrates, we used SEM, which confirmed substantial cell spreading on AAO-NT^PLL^, indistinguishable from the template AAO^PLL^ or the control P^PLL^. Despite similar cell flattening, cells seeded on VANTs appeared tightly anchored to the sheet of nanotubes, suggesting a different morphological adaptation compared to AAO^PLL^ or P^PLL^. SEM imaging suggests a tendency of cells to hook in the nanotube layer, with the development of membrane/substrate junctions not detected in cells grown on the control surfaces (fig. 5).

We documented that the NNS layer of BaTiO_3_ does not impair cell cycle progression, suggesting that the change in cell cycle documented in this study depends on the nano-topography of the VANTs rather than their surface chemistry. The roughness of all substrates was evaluated by AFM. P sample shows the flatter surface with a root mean square Rq = 8.08±0.84 and maximal peak to peak height Zpp = 59.31±7.25 (N = 6). While AAO exhibits a relative flat surface with 200 nm empty pores and a centre-to-centre distance of 300 nm [25] (Rq = 15.65±4.70 nm, Zpp = 143.12±42.55 nm, N = 6), AAO-NT is characterised by BaTiO_3_ nanotubes protruding from the pores, resulting in an increase of roughness (Rq = 28.02±5.66 nm, Zpp = 236.00±58.31 nm, N = 6). There is a significant difference in their surface roughness (p<0.05). The NNS layer of BaTiO_3_ is characterised by the highest roughness (Rq = 229.00±51.60, Zpp = 1354.14±355.18, N = 6), with a value of Rq = 44.63±19.00 and Zpp = 289.17±134.92 (N = 6) within the single grain (scanned areas 0.5×0.5 µm^2^). All together, the results of these experiments lead us to exclude any role by the template, the material itself and the roughness, leading us to postulate that the nano-structuration of the VANTs plays a pivotal role. Nanotopography confers to the surface not only an increase in roughness but also an ordered nanostructuration. Our hypothesis is that the regular nano-topography of VANTs increases the availability of surface area per unit of volume and facilitates recruitment and adsorption of PLL (even if the material is unpoled). PLL is a poly amino acid that promotes cell adhesion through its interaction with the negatively charged ions of the cell membrane. By recruiting PLL, the nano-structured AAO-NT^PLL^ presents to cells a high number of positively charged sites per unit of volume, which strongly promote cell adhesion, reproducing the nanotopography of physiological environments such as the extracellular matrix. Compared to P or AAO^PLL^ or NNS^PLL^, AAO-NT^PLL^ could offer a 3D scaffold in which the number of sites available for cell binding would increase to several orders of magnitude. Similarly, by using nanofibers designed to present to cells the neurite-promoting laminin epitope IKVAV at nearly van der Waals density, Silva et al. amplified the epitope density relative to a laminin monolayer by a factor of 10^3^ and reported a strong inhibition of astrocyte proliferation [26]. This mechanism could explain the strong attachment of the fibroblasts to the AAO-NT^PLL^ surface. In order to confirm this hypothesis, we evaluated the cell adhesion strength to the substrate. Mechanical cell detachment by US was very poorly effective on cells cultured on AAO-NT^PLL^, which are very strongly anchored to the substrate compared to P, P^PLL^, AAO^PLL^ or NNS^PLL^ (fig. 8). A strong cell attachment to the surface could be related to a decrease in cell proliferation. It is known that size, shape and adhesion are determinants of migratory, proliferative and differentiation behaviour of anchorage-dependent cells. It has been reported that if the anchorage extent is very limited (i.e. attachment of round cells without spreading), the cells usually do not survive. At intermediate adhesion strength, the cells are most active in migration and proliferation. If the adhesion sites are well developed, the cells tend to skip the proliferation phase and enter the differentiation program [27]. A similar result was achieved by Deligianni et al., who performed behaviour studies of cells seeded on hydroxyapatite, and found greater adhesion strength and reduced proliferation in cells seeded in rougher hydroxyapatite surfaces [28]. Furthermore it is well established that certain specific cells types, including osteoblasts and fibroblasts, exhibit reduced cell proliferation when seeded on rougher substrates [29], [30]. Thus based on the reported literature and findings of the present study, it is possible to infer that cell differentiation or proliferation can be a direct effect of implanted materials, which are able to influence cell behaviour by affecting cell cycle. In essence the results of the present study have documented reduced cell cycle progression of NIH-3T3 cells grown on VANTs of BaTiO_3_. This effect cannot be attributed to any cytotoxic effect or impaired cell spreading, instead, it seems to depend on the strong improvement of mechanical adhesion of cells to the substrate, promoted exclusively by the VANTs.

One of the most important causes of the implant failure is loss of material-tissue interface due to fibrous encapsulation, where the predominant tissue forming cell phenotypes is fibroblastic [16]. Therefore, materials able to reduce cell adhesion and proliferation of specific cell types, like fibroblasts, are of interest for biomedical applications. Other biological phenomena relating to encapsulation such as the expression of collagen-relating genes and the secretion of collagen will be investigated in future works to assess the translational potential of this finding to medical implant design.
